# Peridural Anesthesia and Cancer-Related Survival after Surgery for Pancreatic Cancer—A Retrospective Cohort Study

**DOI:** 10.3390/clinpract11030070

**Published:** 2021-08-18

**Authors:** Andrea Alexander, Nadja Lehwald-Tywuschik, Alexander Rehders, Stefanie Rabenalt, Pablo E. Verde, Claus F. Eisenberger, Nina Picker, Wolfram Trudo Knoefel, Peter Kienbaum

**Affiliations:** 1 Department of General, Visceral and Pediatric Surgery, University Hospital and Medical Faculty of the Heinrich-Heine-University Düsseldorf, 40225 Düsseldorf, Germany; andrea.alexander@med.uni-duesseldorf.de (A.A.); Nadja.Lehwald-Tywuschik@med.uni-duesseldorf.de (N.L.-T.); Rehders@med.uni-duesseldorf.de (A.R.); eisenbergercf@kliniken-koeln.de (C.F.E.); nina.picker@rkm740-klinik.de (N.P.); 2 Department of Anesthesiology, University Hospital and Medical Faculty of the Heinrich-Heine-University Düsseldorf, 40225 Düsseldorf, Germany; stefanie.rabenalt@med.uni-duesseldorf.de (S.R.); Peter.Kienbaum@med.uni-duesseldorf.de (P.K.); 3 Coordination Center for Clinical Trials and Biostatistics, Heinrich-Heine-University Düsseldorf, 40225 Düsseldorf, Germany; pabloemilio.verde@hhu.de

**Keywords:** oncological outcome, staging, recurrence, pancreatic adenocarcinoma

## Abstract

Background: In patients with prostatic and breast cancer the application of peridural anesthesia (PDA) showed a beneficial effect on prognosis. This was explained by reduced requirements for general anesthetics and perioperative opioids as well as a lower perioperative stress level. The impact of PDA in patients with more aggressive types of cancer has not been completely elucidated. Here, we analyzed the prognostic influence of PDA on overall survival after surgery as primary in patients that underwent radical resection of pancreatic adenocarcinoma. Methods: Records of 98 consecutive patients were reviewed. In 70 of these cases PDA was applied. Patient characteristics such as demographics, TNM stage, and operative data were retrospectively collected from medical records and analyzed. Survival data were analyzed by Cox’s proportional hazard regression model. Results: Overall, no significant prognostic influence of PDA on recurrence or overall survival (*p* = 0.762, Hazard Ratio [HR] 0.884, 95% confidence interval [CI] 0.398–1.961) was found. However, there was a trend towards a longer overall survival (*p* = 0.069, HR 0.394, 95% CI 0.144–1.078) associated with PDA in a subgroup of patients with better differentiation of pancreatic adenocarcinoma. Conclusion: The observation of longer survival associated with PDA in our subgroup of patients with better-differentiated pancreatic carcinomas is in line with previous reports on various other less aggressive tumor entities. Our results indicate that PDA might improve the oncological outcome of patients with pancreatic adenocarcinoma.

## 1. Introduction

Pancreatic adenocarcinoma is a leading cause of cancer-related mortality worldwide [1,2]. Over half of the patients have metastases on presentation, whereas only 15% of patients have resectable disease [3]. Although complete surgical resection is the only curative treatment option for this cancer entity, even after radical surgery prognosis remains poor [4].

Cancer surgery is often associated with systemic release of tumor cells, particularly during the vulnerable perioperative period [5,6]. Hereby, three factors of stress response are considered to impair cellular immunity: the response to surgical trauma, to general anesthesia, and to opioid analgesia [7]. Furthermore, general anesthesia itself is known to impair various immune components such as neutrophil, macrophage, dendritic cell, T-cell, and NK-cell functions [8,9,10,11,12]. In addition, opioids given during anesthesia and for postoperative pain control inhibit humoral and cellular immune response, natural killer cell activity, cytokine expression and phagocytic activity [12,13].

Beneficial effects of regional anesthesia have been demonstrated on the mentioned factors suppressing immune function. Neuroaxial anesthesia decreases the neuroendocrine stress response to surgical tissue injury by blocking afferent noxious input as well as efferent sympathetic transmission [14,15]. Thus, it reduces the need for general anesthesia, minimizes perioperative opioid requirement [16] and improves the short-term perioperative outcome [17,18,19,20].

Accordingly, it has been hypothesized that the incidence of cancer recurrence is decreased after surgery with regional anesthesia and analgesia compared to surgery with general anesthesia and opioid analgesia alone. To date, beneficial effects of regional anesthesia on oncological outcome have been associated with other tumor entities such as prostatic [21], breast [22], bladder [23], colorectal [24] and gastric cancer [25]. Whereas these trials have suggested a positive effect of regional anesthesia on cancer recurrence, others have not found any significant improvement in studies including patients with varying abdominal cancer surgery [26,27]. However, the tumor entities of prostatic and breast cancer, which have mainly been shown to be associated with a reduced recurrence when regional anesthesia was applied [21,22], are characterized by a comparably slow tumor progression, frequently depending on hormone receptor status. To date, the prognostic effect of PDA on pancreatic cancer has not been investigated.

An improved understanding of the effects of perioperative management could lead to better oncological outcomes. Therefore, we hypothesized that the administration of PDA may also improve overall survival in pancreatic adenocarcinoma. 127 consecutive patients with pancreatic ductal adenocarcinoma that had undergone radical tumor resection within five years at our institution were analyzed for overall survival in a retrospective observational study.

## 2. Materials and Methods

All collected data adhered to the guidelines established by the Declaration of Helsinki and has been approved by the ethics committee of the Medical Faculty, Heinrich-Heine-University, Düsseldorf, Germany (2020-848). 

This is an observational retrospective study to analyse overall survival as a primary endpoint after a median follow-up time of 17.26 months. Clinical data were collected from patients’ medical records, compiled into an Excel-file database and analyzed retrospectively. The following data were collected: demographic parameters (age, gender, ASA-score), tumor characteristics (T-stage, N-stage, grading), treatment characteristics (surgical procedures, type of anesthesia, operating time, blood transfusion).

### 2.1. Anesthesia

All patients underwent surgery under general anesthesia (GA). GA was induced with intravenous sufentanil (0.1–0.5 µg kg^−1^), thiopental (3–7 mg kg^−1^), cisatracurium (0.1 mg kg^−1^) and maintained with sevoflurane (endtidal concentration 1.2–2.8 Vol.%). Additional boli of sufentanil were administered as required.

In addition to GA, thoracic peridural anesthesia (PDA) is usually recommended for patients undergoing complex viscero-surgical procedures at our institution. Patients who did not give their consent, patients with coagulopathies, local infections or other contraindications, such as a high probability to undergo complex vascular reconstruction with consecutive anticoagulation, were excluded from PDA. Prior to induction of GA a peridural catheter was inserted at the thoracic level (T6–T10). Typically, a bolus of 15–150 mg of ropivacaine (0.375–0.75%) was given initially. During surgery PDA was maintained by a continuous infusion of ropivacaine (7.5–50 mg/h, 0.375%), supplemented by peridural sufentanil (total dose 15–135 µg) at the discretion of the attending anesthesiologist.

### 2.2. Surgical Procedures

Surgery always aimed at complete tumor resection without microscopic residues. Surgical procedures included partial pancreatoduodenectomy, and distal and total pancreatectomy. Partial pancreatoduodenectomy was usually performed as a pylorus-preserving procedure with a three-loop reconstruction. Lymphadenectomy routinely included clearance of the peripancreatic, hepatoduodenal, celiac and interaortocaval lymph nodes. All procedures were carried out by a transverse laparotomy.

### 2.3. Postoperative Pain Management

Postoperative analgesia was monitored by an interdisciplinary team of surgeons and anesthesiologists. All patients, except those with an established allergic history or intolerance, received metamizole at a rate of 1 g every 6 h. As a routine, patients with peridural catheters received continuous peridural ropivacaine 0.2% with infusion rates of 4–12 mL/h. In case of insufficient analgesia for mobilisation or respiratory physiotherapy patients received intravenous piritramide up to 30 mg per day. Patients without peridural catheters were treated with intravenous piritramide through patient-controlled analgesia or per physician’s order in case of poor compliance. Furthermore, all patients received intravenous paracetamol up to 4 g per day and/or etoricoxib 60 to 120 mg per day, if required. Peridural catheters were removed in case of inadequate function, suspected infection, if no longer required by the patient or at the latest on POD 7.

### 2.4. Follow-Up

Follow-up was performed every 3 months for the first two years after surgery, every 6 months until 5 years after surgery and every year thereafter. Follow-up examinations included a physical examination and medical history, an abdominal ultrasound, a complete laboratory work-up, and a chest and abdominal computed tomography. If patients were not followed at our institution, a study nurse contacted the referring physician at the same time intervals mentioned above to collect the data.

### 2.5. Statistical Analysis

Descriptive statistical analysis and graphing were performed using MS Excel from Microsoft (Redmond, Washington, DC, USA) and JMP 14.1 from SAS Institute Inc. (Cary, NC, USA). All results were expressed as mean ± standard deviation. Statistical significance was determined by Student’s *t*-test and the χ^2^ test for two-by-two frequency tables.

A Cox’s proportional hazard regression model was applied to investigate the association between overall survival and perioperative administration of PDA, adjusted by the following covariates: age, vascular invasion, sex, operation time, T-stage, N-stage, grading, administration of adjuvant chemotherapy and ASA-score. To achieve a parsimonious model, a model selection procedure was established by choosing the regression model with minimum AIC (Akaike Information Criterion) [28]. The model class runs from the model including all covariates to the model with a constant term. Second order interactions were further analyzed between the selected variables in the model. The stability of this model selection process was validated by taking 500 bootstrap samples and by repeating the full selection process in each sample [29]. Results of the model are presented in terms of hazard ratios and their 95% confidence intervals calculated from the Cox’s regression model. We tested the crucial assumption that the hazards are proportional over time by using the Schoenfeld residuals.

We performed a retrospective sample size determination for a two-sided log-rank test, which is equivalent to the test calculated from the proportional hazard Cox’s regression. We took a significance level of 5% two-sided and corrected by Bonferroni for sub-group analysis, i.e., the final significance level for the sample size determination was 2.5%. We utilized a power of 80% and expected HR = 0.40. Then, when the sample size in each group is 60, with a total number of events required, E, of 45, a 0.025 level two-sided log-rank test for equality of survival curves will have 80% power to detect the difference between two curves with a constant hazard ratio of 0.4. In our data we have a total of *n* = 96 patients, 24 less patients than required, and E = 51 events, 6 more events as indicated in the sample size determination. Clearly, this is a retrospective explorative study, but the number of events and the number of patients is close to the required number by the sample size determination.

The statistical analysis was performed using the statistical software R version 4.0.2 (R Core Team, Vienna, Austria, 2020) [30]. We used reporting tools based on the standards of replicable research using the R’s package “knitr” [31]. The analysis based on the proportional hazard Cox’s regression and the estimation of the C-statistics was performed with the R’s package “survival” [32].

## 3. Results

Medical records of a total of 127 consecutive patients with pancreatic ductal adenocarcinoma that had undergone radical tumor resection at our institution were analyzed in a retrospective observational study. 19 patients (15%) with residual tumor (R1 or R2 resections) as well as 9 patients (6.3%) who died perioperatively (60 days) were excluded. One of them died perioperatively after R1 resection. Accordingly, 100 patients remained in the study for further analyses.

During follow-up, two patients died for other reasons than tumor recurrence and were excluded from the prognostic analysis, which is based on the remaining 98 patients.

Our patient collective consisted of 48 (49%) male and 50 (51%) female patients, the median age was 65 years (range 41–85 years).

Patient and tumor characteristics are summarized in Table 1.

The majority of the patients (62.2%) had a reduced physical health status with an ASA score of 3 or more. Most patients were diagnosed at an advanced tumor stage. 89.8% presented with large primary tumors (pT3 or pT4) whereas 76.5% had lymph node involvement (pN1). 53.1% of the patients had poorly differentiated primary tumors.

### 3.1. Surgical Procedure

89 patients (90.8%) underwent partial pancreatoduodenectomy, 5 patients (5.1%) had distal pancreatectomy, and 4 patients (4.1%) required total pancreatectomy (Table 2).

In 26 patients (26.5%) infiltration of neighbouring large vessels—usually the portal vein or the superior mesenteric vein, respectively—was found. In order to achieve clear resection margins, vascular resection with subsequent reconstruction was performed in all of these cases. One patient received an additional reconstruction of the celiac trunk, and another patient had a segmental resection of the common hepatic artery, which was reconstructed by termino-terminal anastomosis. The mean duration of surgery was 413 min with a range of 210–755 min and a standard deviation of 103 min. For statistical analysis patients were divided into two groups with duration of surgery of either <420 min (59 patients) or >420 min (39 patients). The majority of patients (90%) showed a limited intraoperative blood loss with a maximum transfusion of 4 packed red blood cell concentrates. Four patients received 5–8 red blood cell concentrates and four patients needed more than 8 transfusions of packed red blood cell concentrates during surgery (Table 2).

### 3.2. Anesthesia

In 28 (28.6%) of the patients, surgery was performed under GA, whereas 70 (71.4%) received GA + PDA. All patients received intravenous sufentanil during the procedure. If required to attain sufficient analgesia, additional boli of sufentanil were given peridurally. There was a wide range of overall intraoperative dosages of opioids varying between 15 and 150 µg (data not shown).

### 3.3. Adjuvant Therapy

All patients were assessed by a multidisciplinary tumor board consisting of oncologic surgeons, oncologists, radiotherapists, pathologists and radiologists. According to the recommendation of the tumor board, 79 patients (80.6%) received adjuvant chemotherapy (Table 2).

### 3.4. Survival Data

Mean follow-up time after pancreatic resection was 17.26 months, ranging from 3 to 66 months. Overall 1-, 3- and 5-year survival rates after resection were 72.29 months and 17%, respectively, without differences between groups (GA only vs. PDA + GA).

### 3.5. Prognostic Parameters

The assessment of parameters with potential impact on prognosis included T-stage, N-stage, grading, operating time, age, ASA classification and amount of red blood cell concentrates transfused. With respect to clinic-pathological parameters, our results did not show any statistical significance between the two groups (PDA + GA vs. GA only) (Table 3).

Survival data analyzed with respect to prognostic data are summarized in Table 4. Grading was found to be of prognostic significance at univariate analysis. Patients with well-differentiated primary tumors (G_1–2_) showed a mean overall survival of 35.15 months compared to 22.86 months in patients with poor tumor differentiation (*p* = 0.030). Tumor involvement of the resected lymph nodes (pN1) was also associated with worse prognosis, but the difference in overall survival time (38.48 vs. 23.25 months) did not reach statistical significance (*p* = 0.057).

**Figure 1:**
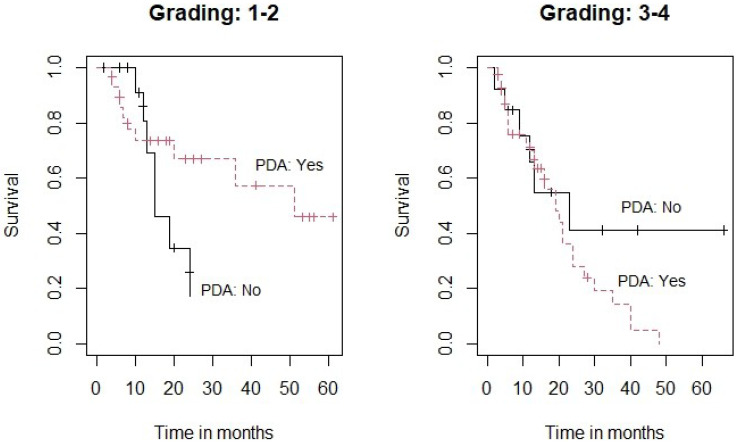
Subgroup analysis for survival of the administration of PDA by grading. Left panel: grading score 1 to 2. Right panel: grading score 3 to 4.

In this analysis the application of PDA in addition to GA was not found to be a significant prognostic parameter concerning overall survival (Table 4). However, since the grading showed a certain impact on the results, we decided to perform a subgroup analysis investigating the potential impact of PDA on the grading subgroups G_1–2_ vs. G_3–4_ (Figure 1).

Figure 1 demonstrates Kaplan-Meier survival curves for the two subgroups. Subgroup analysis showed that the effect of PDA was not statistically significant for the subgroup of patients with less differentiated tumors (G_3–4_) (*p* = 0.191) (Right panel). However, there was a clear trend towards an improved survival when PDA was used in the subgroup of patients with a better tumor differentiation (G_1–2_) (*p* = 0.069) (Left panel).

## 4. Discussion

The use of peridural anesthesia and its association with improved survival after cancer surgery has been described for different tumor entities in multiple retrospective studies [33,34,35]. Initial studies on the role of PDA on cancer outcome have stated a reduction in tumor recurrence and metastases of breast and prostatic cancer when surgery was performed under general anesthesia combined with regional analgesia. This prognostic impact was attributed to a lesser impairment of immune surveillance, caused by a decrease of the neuroendocrine stress response to surgical trauma [21,22].

Both prostate cancer as well as breast cancer are frequently dependent on hormone receptor status and are characterized by a rather prolonged disease course. However, to date there is less evidence on the prognostic impact of peridural analgesia in malignant entities with a more aggressive tumor biology. Its effect for pancreatic cancer remains mostly unexplored. To the best of our knowledge, this is the first study investigating the role of peridural anesthesia on the outcome of patients with pancreatic adenocarcinoma, which is known for its very aggressive biological potential. Reported 5-year survival rates after surgery range between 4–25% [36,37,38]. In our collective the 5-year survival time was calculated at 17%, which is in line with other reports, considering the large proportion of patients with locally advanced disease and lymph node involvement.

Collectively, our study revealed no significant prognostic benefit when PDA was administered, yet a subgroup analysis of patients with better tumor differentiation (G_1–2_) showed that PDA in these patients was correlated with an improved overall survival. In contrast, no prognostic impact was observed in patients with poorly differentiated tumors.

Interestingly, further studies have reported positive prognostic effects associated with PDA in certain subgroups. In a study analyzing the effect of PDA in patients with colorectal cancer, a prognostic benefit for a subgroup of patients with rectal cancer was found [39]. Furthermore, an improvement in survival in patients receiving peridural anesthesia for colorectal cancer surgery was shown [40], but solely for metastasis-free patients for 1.5 years. Likewise for colorectal cancer, a potential benefit for patients > 64 years was observed [41], and prolonged survival, yet no impact on cancer recurrence [24] was described. This corresponds to our findings. The effect may depend on the specific type, location or aggressiveness of the tumor.

Generally, the data reported on this subject is characterized by rather controversial results. In addition to prostatic and breast cancer, several analyses could show a significant association of epidural anesthesia and improved overall survival for patients with gastric cancer [25,42,43,44,45]. A study including patients with bladder cancer revealed an increased five-year survival in patients who received regional anesthesia, although this association was not significant [46]. Investigating the effect of perioperative dexamethasone on survival after resection of pancreatic adenocarcinoma, Call et al. found increased survival when PDA was used [47].

In contrast, no benefit of PDA on overall survival or cancer recurrence was found in patients with colorectal [48], prostatic [49] or bladder cancer [8,50]. Evaluating patients with major abdominal surgery for cancer, further studies [8,26,27,45] could not observe any significant influence of anaesthetic technique on patients’ outcome, either. Yet, in these collectives, the patients had various surgical procedures including gastrectomy, pancreatectomy, colectomy, hepatectomy, cystectomy, nephrectomy or prostatectomy. Thus, a potential effect of PDA regarding cancer recurrence or overall survival might be multifactorial and therefore inhomogenous.

In summary, our findings, as well as several of the above-mentioned results of previous studies, suggest that PDA might have a favourable effect on long-term prognosis of patients with solid malignant tumors. However, it remains unclear if this effect is also present in patients with more aggressive and poorly differentiated primary tumors. Presumably, these patients could benefit from a reduced preoperative stress response and opioid requirements.

The limitation of our study includes the retrospective observational nature of the analysis. Moreover, ropivacaine and sufentanil doses were selected at the discretion of the attending anesthesiologist and were not administered according to a standardised protocol. The received amount of PDA may therefore differ between patients.

## 5. Conclusions

Overall, we could not show any significant prognostic benefit. Consistent with previously published data, which show prognostic impacts of PDA within subgroups, our findings suggest an improved survival for the subgroup of patients with better tumor differentiation (G_1–2_). Therefore, patients with better tumor differentiation of pancreatic adenocarcinoma may benefit with respect to overall survival.

The assumption that regional anesthesia may have an effect on outcome after oncological surgery is supported by numerous retrospective clinical studies.

To date, a variety of observational clinical data have been released—the overall conflicting findings indicate the need for further evidence from large prospective, randomized-controlled trials.

## Figures and Tables

**Table 1:** Patient and tumor characteristics.

Patient and Tumor Characteristics	*n* = 98	
	*n*	%
Gender		
male	48	49
female	50	51
ASA-Score		
ASA 1–2	37	37.8
ASA 3–4	61	62.2
Primary tumor		
pT1–2	10	10.2
pT3–4	88	89.8
Nodal status		
pN0	23	23.5
pN1	75	76.5
Grading		
G1–2	46	46.9
G3–4	52	53.1

ASA: American Society of Anesthesiologists.

**Table 2:** Treatment characteristics.

Treatment Characteristics	*n* = 98	
	*n*	%
Pancreatic resection		
pancreatoduodenectomy (Whipple)	89	90.8
distant pancreatectomy	5	5.1
total pancreatectomy	4	4.1
Vascular reconstruction		
yes	26	26.5
no	72	73.5
Anesthesia		
GA only	28	28.6
PDA + GA	70	71.4
Operating time		
<420 min	59	60.2
>420 min	39	39.8
Adjuvant chemotherapy		
yes	79	80.6
no	19	19.4
Transfusion		
0–4 units	90	91.8
5–8 units	4	4.1
>8 units	4	4.1

GA: general anesthesia; min: minutes; PDA: peridural anesthesia.

**Table 3:** Comparison of clinicopathological parameters in patients with and without PDA.

Clinicopathological Parameters	PDA + GA *n* = 70	GA Only *n* = 28	*p*-Value
T-stage			
pT_1–2_	9	1	
pT_3–4_	61	27	0.170
N-stage			
pN_0_	18	6	
pN_1_	52	22	0.656
Grading			
G_1–2_	29	15	
G_3–4_	41	13	0.275
Operating time			
<420 min.	46	13	
>420 min.	24	15	0.139
Transfusion			
0–4 units	63	25	
5–8 units	6	0	
>8 units	1	3	0.121
Age			
41–55 years	14	6	
56–70 years	30	9	
71–85 years	26	13	0.597
ASA Score			
ASA 1–2	27	10	
ASA 3–4	43	18	0.472

ASA: American Society of Anesthesiologists; GA: general anesthesia; min: minutes; N: node; PDA: peridural anesthesia; T: tumor.

**Table 4:** Results of the three statistical models fitted with the proportional hazard Cox’s regression. In each model, the risk factors are reported with the estimated hazard ratio and its 95% confidence intervals. At the bottom line the AIC (Akaike Information Criterion) is reported. A lower AIC indicates a better prognostic value of the model. The bootstrap probabilities that a risk factor is included in a stepwise variable selection are presented in the last column.

	Model 1: All Risk Factors				Model 2: PDA Only				Model 3: PDA Subgroups				Bootstrap Probability (%)
Risk Factor	HR	Lower 95% CI	Upper 95% CI	*p*-Value	HR	Lower 95% CI	Upper 95% CI	*p*-Value	HR	Lower 95% CI	Upper 95% CI	*p*-Value	
PDA yes	0.8839	0.3984	1.961	0.7615	0.9902	0.5255	1,866	0.976					11.73
Age ≥ median	1.1978	0.6368	2.253	0.5756									3.8
Sexm	0.7419	0.4126	1.334	0.3185									8.53
T.group 3 + 4	1.4276	0.4882	4.174	0.5155									7.44
Nyes	1.7516	0.8220	3.733	0.1465									8.83
G.group 3 + 4	0.5461	0.2925	1.020	0.0576					1.5354	0.5057	4.662	0.4492	15.64
ASA.group 3 + 4	1.5002	0.7727	2.913	0.2309									9.06
Chemotherapieyes	0.5799	0.2847	1.181	0.1333									8.72
Vascular.invyes	1.0779	0.5291	2.196	0.8363									5.30
OP.time > 420	1.2643	0.6427	2.487	0.4969									5.56
Opiate.iv > 50	1.2021	0.5651	2.557	0.6327									3.65
G.group < 3: PDAyes									1.8108	0.7435	4.410	0.1911	11.73
G.group 3 + 4: PDAyes									0.3937	0.1438	1.078	0.0697	11.73
AIC	387.3443				381.0751				375.2419				

## Data Availability

Supplementary data presented in this study are available on request from the corresponding author.

## Abbreviations

ASA	American Society of Anesthesiologists
GA	general anaesthesia
NK cells	natural killer cells
PDA	peridural anesthesia
POD	postoperative day
T cells	thymus cells
